# Absorption, Scintigraphy, Efficacy and Tolerability of Short-Term Calcium Carbonate Supplementation in Healthy Subjects

**DOI:** 10.7759/cureus.87642

**Published:** 2025-07-09

**Authors:** Gaurav K Jain, Nazeer Hasan, Nilesh Chandra, Abha Mishra

**Affiliations:** 1 Department of Pharmaceutics, Centre for Advanced Formulation Technology, Delhi Pharmaceutical Sciences and Research University, New Delhi, IND; 2 Department of Pharmaceutics, School of Pharmaceutical Education and Research, Jamia Hamdard, New Delhi, IND; 3 Division of Discovery Research, Indian Council of Medical Research Head Quaters, New Delhi, IND; 4 Department of Physiology, Jodhpur Institute of Engineering and Technology, Medical College and Hospital, Jodhpur, IND; 5 Department of Physiology, Delhi Diabetes Clinic, New Delhi, IND

**Keywords:** absorption, bone density, bone marker, calcium carbonate, efficacy, in vitro dissolution, parathyroid hormone, pharmacokinetics, scintigraphy, tolerance

## Abstract

Orally administered calcium carbonate tablets are commonly prescribed as a calcium supplement, and their short-term use is popular among the healthy population. However, being a nutritional supplement, the in vitro properties and clinical efficacy of calcium carbonate supplements are sometimes compromised. The present study aimed to assess the absorption, in vivo dispersion, efficacy and tolerance of Gemcal DS tablet following its short-term use for four weeks. Post-dosing, a gradual rise in serum calcium concentration was observed and the peak increment in serum calcium (4.13±0.38 µg/mL) was reached at four hours. The bioavailability, determined as the area under the curve for six hours (ΔAUC_0-6_) of serum calcium, was 38.2±4.8 µg/mL/hour.

Scintigraphy images showed that the disintegration of the study product initiated within 15 minutes in the stomach, with the radioactive trail suggesting complete dispersion within four hours in the small intestine. No intact tablet was observed in the small intestine or the large intestine. An increase in mean serum calcium (~3%) and procollagen type 1 N-terminal propeptide (P1NP) (~13.2%) was observed post-treatment.

In contrast, a decrease in parathyroid hormone (PTH) levels was noted. Dual-energy X-ray absorptiometry (DEXA) scan results revealed an increase in bone density from 1.1968±0.05 (baseline) to 1.2115±0.06 g/cm^2^, post-treatment. T-scores were also improved in all the subjects, except 1 subject whose T-score remained the same. The Gastrointestinal Symptom Rating (GSR) score at the end of the study (0.42±0.62) was not significantly different (p>0.05) from the baseline GSR score (0.33±0.54), indicating that the treatment was safe and tolerable. In conclusion, Gemcal DS tablets produced an appropriate pharmacokinetic and pharmacodynamic response and could be recommended for short-term use in the healthy population.

## Introduction

The present clinical study assesses the absorption, scintigraphic dispersion, short-term efficacy and tolerance (ASSET) of calcium carbonate in healthy individuals. Calcium is one of the essential macronutrients that plays an important role in maintaining human health [1]. The calcium requirement of the body varies throughout life, and the demand is particularly high during the growth phase, puberty, pregnancy, lactation, old age, etc [2]. To balance the demand and maintain homeostasis, a supplementary intake of calcium is anticipated [3]. Thus, the dietary calcium requirements during the demanding stage are considerably high and, in many instances, the required amount of calcium is not absorbed, and the calcium balance becomes negative [4].

The absorption of calcium from the gastrointestinal tract (GIT) is site-specific, carrier-mediated and pH-dependent. Furthermore, efficacy is also limited by the intra-luminal presence of dietary components (such as oxalate, phytate, fat, and fibre), disease states (including vitamin D deficiency), medications (such as antacids and proton pump inhibitors), and age [5,6]. All these factors limit the oral absorption of calcium, leading to its low oral bioavailability (~20-30%). Despite this, oral calcium products, particularly tablets are widely prescribed for long-term use in correction of hypocalcemic states [7] and in variety of diseased conditions including the management of hypertension [8], correction of hypocalcemic states, treatment and prevention of osteoporosis [9], prevention of gastroesophageal reflux disease [10] and as phosphate binders in chronic renal failure [11]. Among calcium salts, calcium carbonate, with its higher elemental calcium content and lower cost, is generally preferred [12]. Calcium carbonate tablets are also available commercially as over-the-counter supplements for short-term use by the healthy population in India [13]. According to a recent report, the market size of calcium carbonate is estimated at USD 55.48 billion in 2025 and is expected to reach USD 89.77 billion by 2033 [13].

The practice of generic drug substitution is widespread in India. Before generic drugs are marketed, they are required to meet rigid standards determined by the CDSCO [14]. However, calcium carbonate is classified as a nutritional supplement, and not a drug; therefore, there is almost no regulation of its manufacture. Carr and Shangraw (1987) performed disintegration and dissolution of 32 commercial brands of calcium carbonate tablets and showed that 25 brands failed to meet the requisite regulatory criteria [15]. Kobrin and group (1989) also reported two cases where a commercial brand of calcium carbonate tablets appeared to be clinically ineffective [16]. There are a few possible explanations for these ineffective calcium carbonate products. Calcium carbonate requires low pH (acidic conditions) for optimal disintegration and dissolution. Patients with rapid stomach transit or achlorhydria might exhibit slow dispersion of tablets. Further, the tablet should disintegrate in the stomach so that calcium is liberated at the target site for enhanced absorption. Moreover, over-compression of tablets to achieve small-sized tablets, formulations with low disintegrating agents or use of acid-insoluble coating agents may also prevent disintegration and dissolution of calcium carbonate in the stomach [17]. Thus, assessment of dispersion, kinetics and quality of commercial calcium carbonate tablets is imperative.

Though the long-term efficacy and safety of calcium carbonate preparations in disease conditions have been well established [18,19] but no report to the best of our knowledge has assessed the short-term efficacy and safety of calcium carbonate and its absorption in the healthy population. Considering these drawbacks, the ASSET study was planned. Being widely popular, the Gemcal DS tablet was used as a study product. Efficacy was assessed through serum calcium [20], serum parathyroid hormone (PTH) [21], bone marker procollagen type 1 N-terminal propeptide (P1NP) [22] and bone density via dual-energy X-ray absorptiometry (DEXA) scan [23] measurements post-dosing. Tolerance is determined by the well-established Gastrointestinal Symptom Rating (GSR) scale [24].

## Materials and methods

Materials

Study product, Gemcal DS tablet (calcium carbonate 1250 mg, calcitrol 0.25 mcg, vitamin K2-7 100 mcg), was provided by Alkem Laboratories Limited (Mumbai, India). Stannous chloride was purchased from Sigma Aldrich (St. Louis, MO), and technetium-99 m (99mTc) was obtained from Baba Atomic Research Centre (Mumbai, India). All other chemicals and reagents used were of analytical grade and were purchased from CDH Chemicals (India). Scintigraphy and radiolabeling were performed at Dr. Anand Imaging and Neurological Research Centre (New Delhi, India).

Radiolabeling of the tablet

**Figure 1:**
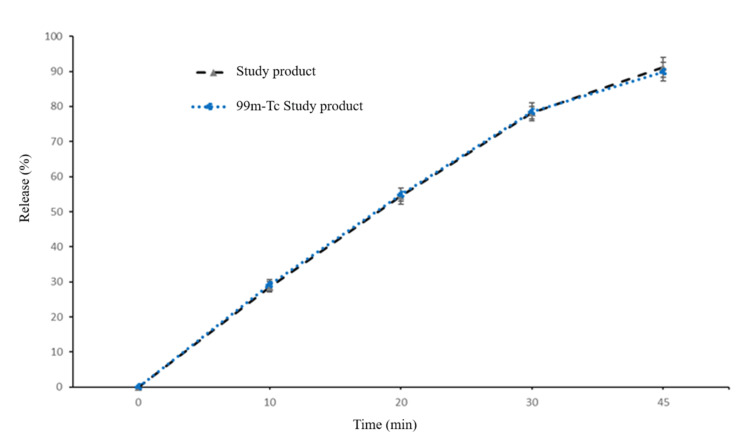
Dissolution profile of non-radiolabeled and 99mTc- radiolabeled study product. Vertical bars represent mean±SD.

For scintigraphy study, the tablets were radiolabeled using 99mTc by the drill and fill method as described previously [25]. Briefly, a radioactive solution was prepared by adding sodium pertechnetate (NaTcO_4_) of about 20 MBq radioactivity into an Eppendorf tube containing 20 µl solution of stannous chloride (2.0 mol/L) in acidified ethanol. A hole in the centre of the tablet was made with the help of a needle and filled with 2 µl of the radioactive solution. The radioactive solution was allowed to diffuse uniformly in the tablet. The remaining space of the hole was filled with lactose and completely sealed with wax. The radiolabeled tablets were measured for radioactive counts using a dose calibrator (PTW Curiementor 2, USA). Labeling efficiency and stability were confirmed by instant thin layer chromatography. The radiolabeling procedure was conducted carefully so that each tablet would have nearly 2 MBq of radioactivity. Similarity in dissolution profile conducted in 900 ml of 0.1 N HCl medium (pH 1.2) at 50 rpm at 37±1℃ of radiolabeled and non-radiolabeled study product validated that the radiolabeling had no impact on dispersion behavior (Figure 1).

Study design

This single-arm, open-label, pilot clinical study was conducted at Dr Anand Imaging and Neurological Research Centre, New Delhi, in collaboration with Delhi Diabetes Clinic, New Delhi, from December 2023 to February 2024. The study was performed in accordance with the principles of the Declaration of Helsinki and the International Conference on Harmonisation Guideline for Good Clinical Practice. The ethical clearance was obtained from the Good Society Ethical Research, New Delhi (GSER/2023/BMR-AP/077).

Subjects

Healthy male subjects who met the inclusion criteria were enrolled in the study. The criteria included an age range of 18 to 60 years, body weight between 60 and 85 kg, and a body mass index (BMI) between 20 and 30 kg/m². Female subjects were excluded due to the scintigraphy procedure. Subjects with a history of renal insufficiency, hypersensitivity to calcium products, or kidney or urinary stones were excluded due to safety concerns. Subjects on diuretics, aluminium salts, thyroid hormones or any other experimental drugs four weeks before the study period were excluded to avoid baseline variations. The details of the study, possible risks and benefits were informed to the subjects. The subjects provided written informed consent before the screening tests and baseline procedures. They were free to withdraw from the study at any time. The demographic details and clinical history of the subjects were recorded after enrolment.

Absorption and scintigraphy imaging

Eight subjects ingested a single dose of radiolabeled study product with a standard meal consisting of two slices of toast with 15 grams of butter, 100 grams of boiled potatoes, two boiled eggs and 200 mL of milk. The subjects were restricted from food or beverages for at least six hours post-dosing. For pharmacokinetic analysis, 5 mL whole blood samples were drawn by direct venipuncture into EDTA tubes at 0, 0.5, 1, 1.5, 2, 2.5, 3, 4 and 6 hours after dose administration. Plasma was separated by centrifugation at 3000 rpm for 10 minutes and stored frozen (-20°C) until assay. The ΔAUC of serum calcium at six hours was calculated by determining the area between the curve and the baseline value below it by trapezoidal integration. Calculations were done considering baseline values as zero. The time to reach the peak serum calcium concentration (T_max_) yielded the number of hours when the serum calcium was maximum. If there was an equal maximal value among consecutive hours, the mean of the time points was used. 

Scintigraphy imaging was done to observe the dispersion pattern of the study product. Sequential scintigraphy images of the abdominal area, each lasting 25 seconds, were taken with a dual-head SPECT gamma camera (Millennium MG, GE Healthcare, US) fitted with a low-energy, high-resolution collimator. The images were captured at the time of dose (0 minute), and 5, 10, 20, 30, 60, 240 or 360 minutes, or until complete disintegration of the tablet, whichever is earlier. The images were analysed using the Genie 4.5 image analysis, and an unpaired two-tailed student t-test was used for statistical analysis.

Efficacy study

The subjects were instructed to continue ingesting the study product (non-radiolabeled) twice daily as a split dose every 12 hours with a standard meal for four weeks. Regular telephonic checks were made to ensure compliance and timely ingestion of the study product. At the end of the study period, the blood samples were withdrawn, and a DEXA scan was performed. Blood estimations and DEXA scan before the start of dosing (baseline values) of the individual subject served as his own control. Blood samples, except for pharmacokinetics, were drawn in the morning in a fasting state.

*S*erum calcium, parathyroid and P1NP

Serum calcium was determined by atomic absorption spectrophotometry [20]. Intact serum PTH was measured using chemiluminescence immunoassay [21] and P1NP by electro-chemiluminescence immunoassay [22]. For measurement of serum calcium and PTH, the samples were sent to Microcare Diagnostics Lab (New Delhi, India), and for P1NP, the samples were sent to THC Lab (Gurgaon, India). All the measurements were done according to the manufacturers' protocols. The inter-assay coefficients of variation were <3.0%

Bone density by DEXA Scan

Whole body DEXA scan was performed at baseline and post-treatment using the Lunar DPX DXA system, Analysis version 13.60 (GE Healthcare, US) at Health Square (New Delhi, India). All patients were supine positioned in the same manner at both DEXA scans. The radiation dose was less than 10 microsieverts. Bone mineral density, signifying the amount of calcium and phosphorus in a particular volume of bone and T-score, a measure of bone density compared to a healthy young adult's peak bone mass, were determined.

Tolerability by the GSR scale

Tolerance was assessed by structured, self-reported responses using the Gastrointestinal Symptom Rating (GSR) scale that assesses the severity of GI symptoms [24]. The scale has 15 items that cover the GI system, including abdominal pain, diarrhoea, reflux, bloating and constipation. The GSR utilises a 7-point response scale to measure subjects’ level of discomfort associated with a given GI symptom, ranging from 1 (No discomfort at all) to 7 (Very severe discomfort). The responses of the subjects were collected at the end of the study period (four weeks), and the mean total score was calculated and compared with the baseline score. 

Statistical analysis

Pharmacokinetics parameters such as ΔAUC, C_max_ and T_max_ were calculated using WinNonlin version 7.0 (Princeton, USA). All the data was presented as the mean±standard deviation (SD). Comparison of numerical variables was done using the paired t-test, and a p-value less than 0.05 was considered statistically significant. All statistical calculations were done using the computer program SPSS V26.0 (IBM Corp., 2019. IBM SPSS Statistics for Windows, Version 26.0. Armonk, NY).

## Results

Absorption and scintigraphy

**Figure 2:**
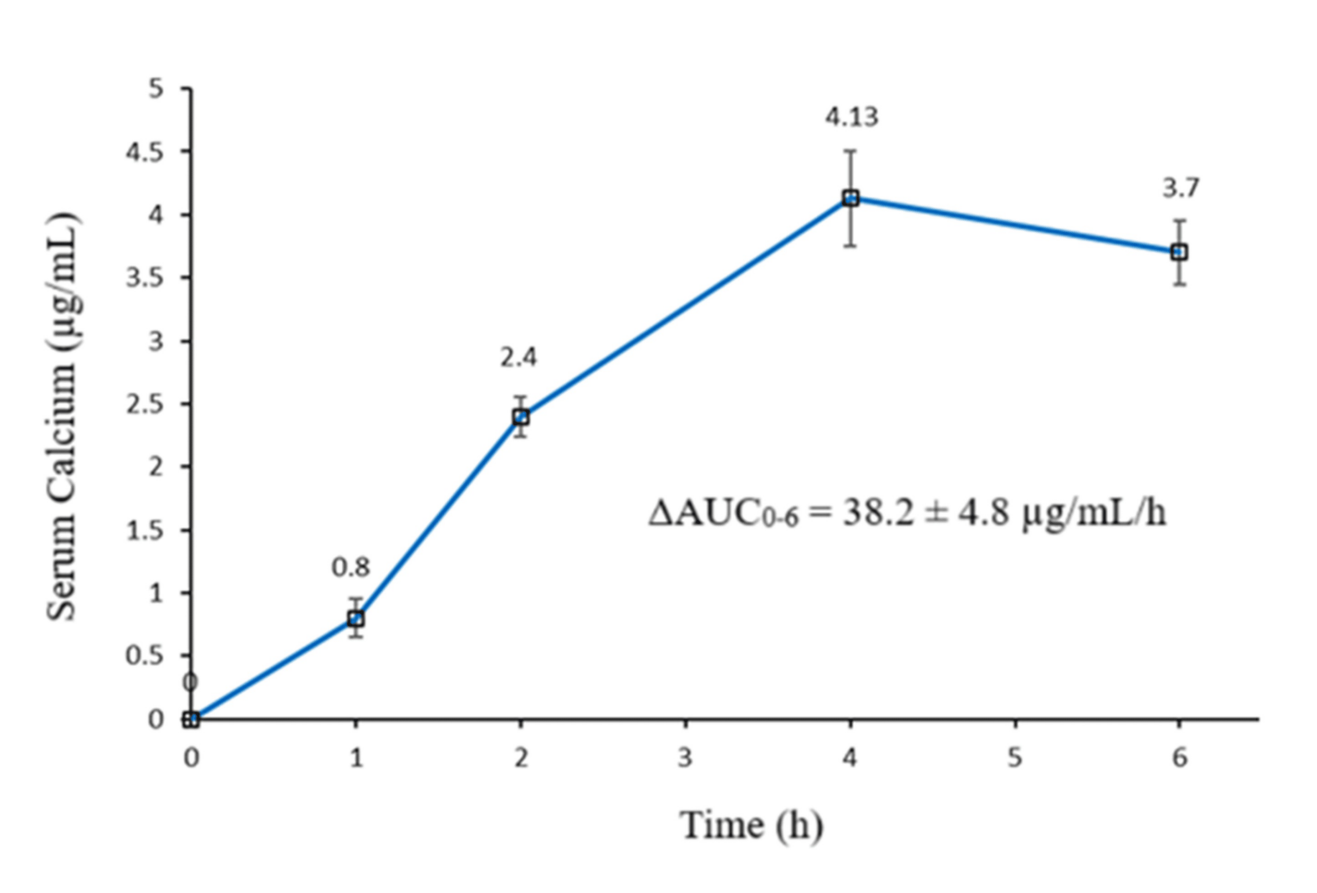
The change in serum calcium concentration from baseline over six hours following oral administration of study product. Vertical bars represent mean±SD.

Figure 2 demonstrates the change in total serum calcium concentration from baseline to six hours following oral administration of the study product. A gradual rise in serum calcium concentration was observed, and the peak increment in serum calcium (4.13±0.38 µg/mL) was reached at four hours post-dose. Thereafter, at six hours, a decline in serum calcium was observed. The ΔAUC_0-6_ of serum calcium was 38.2±4.8 µg/mL/h.

**Figure 3:**
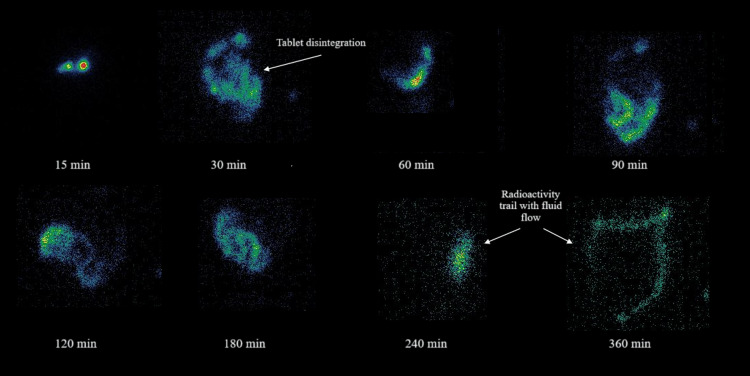
Scintigraphy image of one subject. At 30 min tablet dispersion in stomach is visible. Radioactive trail in subsequent images represent drug dispersed in medium.

Scintigraphy images showed that in all the subjects, the dispersion of the study product initiated within 15 minutes in the stomach. The tablet dispersion was visible in the duodenum from 0.5 hours to 1.5 hours, and complete dispersion of tablets was observed within four hours in the small intestine. No intact tablet was observed in the small intestine and/ or large intestine, and only radioactivity dispersed in the GI medium was observed, indicative of complete tablet dispersion. The representative scintigraphy image of one subject is shown for reference (Figure 3).

Efficacy

**Figure 4:**
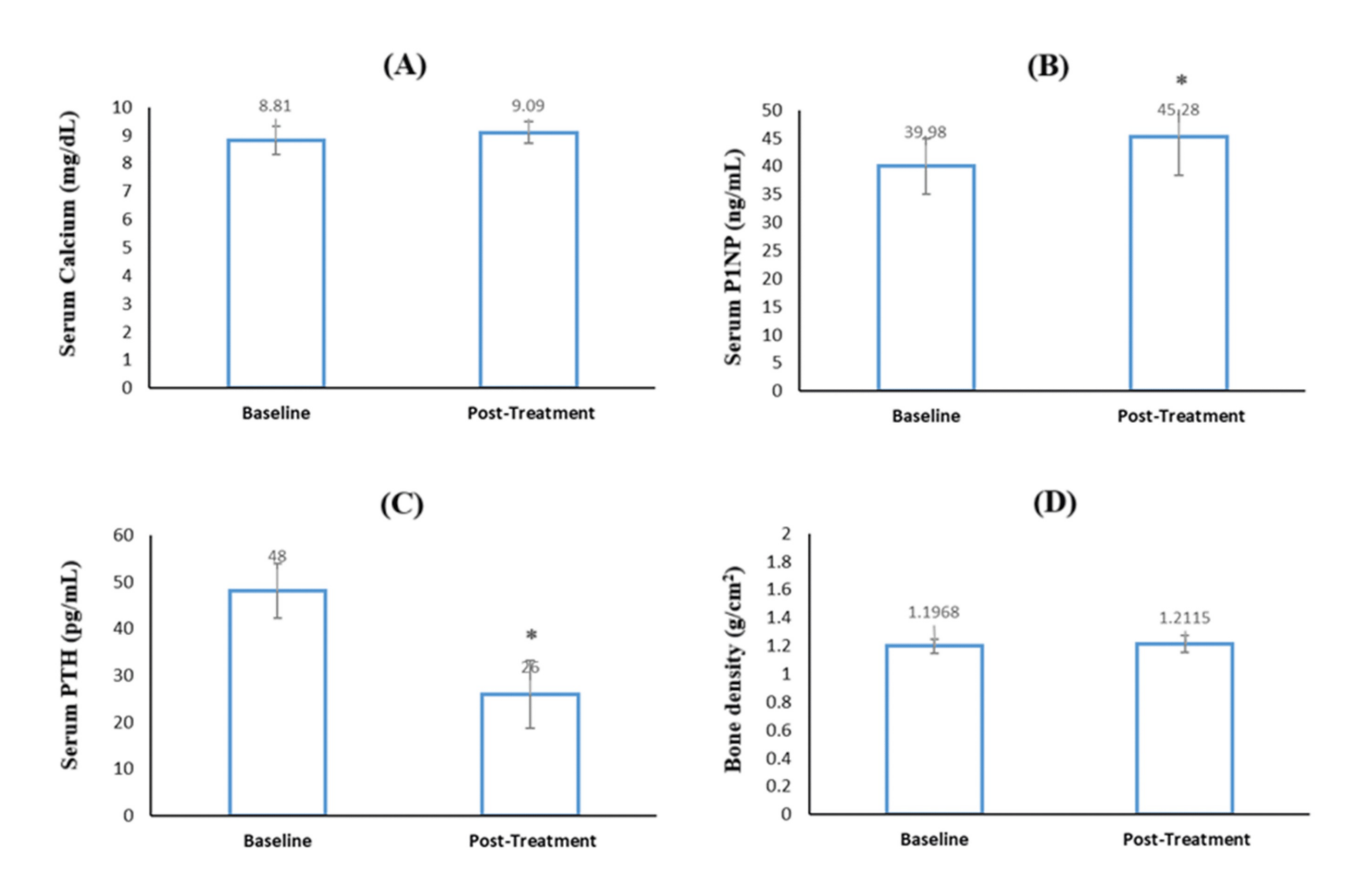
Level of (A) serum calcium, (B) serum P1NP, (C) serum PTH and (D) bone density at baseline and post-treatment following twice daily oral administration of study product for four-weeks. Vertical bars represent mean±SD; * Represents significant difference (p<0.05). Significant p values obtained in 4B (p-value 0.0167) and 4C (p-value 0.0334).

At the end of the study period (four weeks), a modest but important increase in mean serum calcium (~3%) and P1NP level (~13.2%) was observed compared to baseline. The serum calcium increased from 8.81±0.51 to 9.09±0.38 mg/dL (Figure 4A), and P1NP increased from 39.98±4.97 to 45.28±6.90 ng/mL (Figure 4B). Notably, the increase in serum calcium and P1NP post-treatment was observed in all the individual subjects. In contrast, a decrease in PTH levels was observed post-treatment with the study product, and the values were significantly lower than baseline PTH (Figure 4C). Bone density measured by DEXA scan is represented in Figure 4D.

**Figure 5:**
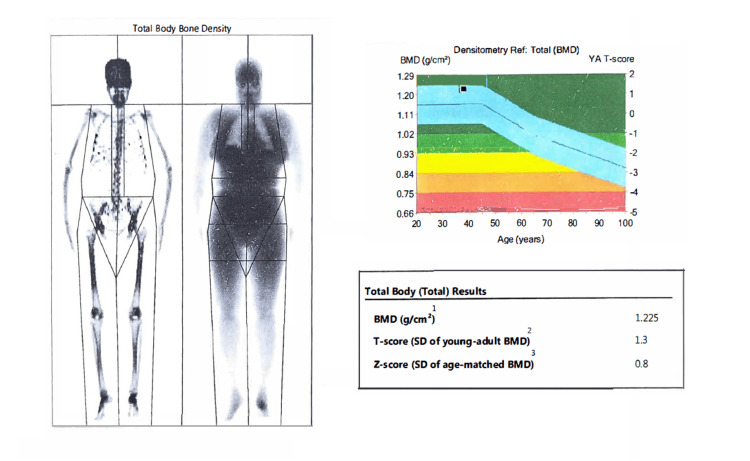
Representative DEXA scan of one subject. The image shows bone mineral density (BMD) and T-score. Representative post-treatment DEXA scan of a subject determined using the Lunar DPX DXA system, Analysis version 13.60 (GE Healthcare, US). The image shows the bone mineral density, the amount of calcium and phosphorus in a particular volume of bone and the T-score, which is the measure of bone density compared to a healthy young adult's peak bone mass.

DEXA scan results revealed an increase in bone density from 1.1968±0.05 (baseline) to 1.2115±0.06 g/cm^2^, post-treatment (Figure 4D). Improved T-scores were observed in all the subjects, sans 1 subject whose T-score remained the same. A representative DEXA scan of one subject is shown in Figure 5. 

Tolerability

No serious adverse events were reported during this study. The GSR score at the end of the study (0.42±0.62) was not significantly different (p>0.05) from the baseline GSR score (0.33±0.54). The constipation was reported by the greatest number of subjects (3/8). However, the reported discomfort was mild and disappeared within one day. The results of the physical examination, vital and laboratory tests remained normal at the end of the study and confirmed the absence of clinically relevant changes in the subjects’ state of health. None of the subjects withdrew from the study because of adverse events.

## Discussion

Short-term use of high-dose calcium carbonate is a popular supplement among the healthy population. Though the long-term efficacy and safety of calcium carbonate in disease conditions have been well established [18,19] but no report to the best of our knowledge has assessed the short-term efficacy and safety of calcium carbonate and its absorption in the healthy population. Assessment of the dispersion of the study product was important since several previous reports demonstrated a lack of bioavailability of commercial calcium carbonate tablets owing to impaired in vitro disintegration and dissolution [15,16]. Thus, for the first time, scintigraphy imaging was conducted to assess the in vivo dispersion of calcium supplements.

The result of the short-term pilot study demonstrated that the study product (Gemcal DS tablet, Alkem Laboratories) is an effective calcium carbonate preparation. Gemcal DS tablet was selected as it is one of the widely sold calcium supplements in India [13]. Being a pilot study, only eight subjects were enrolled. Only healthy males were included because fluctuating estrogen levels in females can significantly influence calcium absorption [26]. Since calcium supplements are recommended with meals, therefore to we instructed subjects to consume the study product with a standard meal. Post-treatment assessments were done after four weeks, and baseline values of the same individual served as control.

Absorption studies showed a gradual rise in serum calcium concentration over period of four hours, upon oral administration of the study product. The peak increment in serum calcium concentration was reached at four hours post-dose. The results were in agreement with the previous studies where a similar rise in serum calcium was observed upon oral administration of calcium supplements [17,27,28]. Calcium absorption from the product has been shown to be affected by several variables, including the type of population, the presence or absence of a co-administered meal, the size of the dose and in vitro disintegration and dissolution properties of the product [20]. Our C_max_ values were higher than those reported by Heller HJ et al. 1999 [27] but lower than those reported by the same group in the year 2000 [28]. Heller and group determined the kinetics of calcium carbonate in healthy women [27], and the same group in 2000 determined kinetics in postmenopausal women [28], which might result in differences in calcium absorption levels since demand for calcium in postmenopausal women would be higher [29]. Further, a co-administered meal might have an effect on calcium absorption, resulting in differences in C_max_ values as observed. Possibly, the interactions between food macromolecules and calcium particles might be responsible for the higher T_max_ (four hours) observed in our study compared to previous reports (T_max_, two hours-three hours) [6,7].

In an earlier report by Wiria MSS et al. (2020), superior calcium absorption from calcium carbonate was reported [17]. However, they used an effervescent tablet, which was first dissolved in water, and the solution was then drunk immediately. The result underscored the importance of disintegration and dissolution in the absorption of calcium carbonate. It is documented that an acid medium is necessary for optimal disintegration and dissolution of calcium carbonate tablets. Once tablets pass through the stomach, the disintegration or dissolution will be impaired in the alkaline medium of the remainder of the GI tract [13,16].

Interestingly, scintigraphy images showed that in all the subjects, the disintegration of the study product initiated within 15 minutes in the stomach and the radioactive trail suggested complete dispersion within four hours in the small intestine. No intact tablet was seen in the small intestine or large intestine. The complete disintegration of the study product in the upper GI might explain the plausible reason for the lowest deviation in C_max_ and the highest AUC0-6 values observed for our study product, despite lower C_max_. The adequate disintegration and dissolution of the study product in vivo also emphasised the quality of the product. The quality of calcium carbonate is important because it is classified as a nutritional supplement, not a drug, and thus, there is almost no regulation of its manufacture. The complete dispersion of the study product at the calcium absorption site in the GI would most likely be responsible for enhanced serum calcium and P1NP levels observed at the end of the short study period (four weeks). The increase in P1NP levels indicates the formation of new bones. This indirectly translates into enhanced calcium transport to bones from serum owing to higher serum calcium [22]. Considering physiologically more meaningful, the per cent change in PTH was determined rather than the absolute change. PTH levels decrease significantly post-treatment, justifying an increase in serum calcium. Further, the results of the DEXA scan were in corroboration with the study findings and increased bone density and T-scores were observed post-treatment.

As expected, being a short-term treatment, the GSR score at the end of the study was not significantly different (p>0.05) from baseline GSR scores. The results of the physical examination, vital and laboratory tests, remained normal at the end of the study and confirmed the absence of clinically relevant changes in the subjects’ state of health. None of the subjects withdrew from the study because of adverse events.

There are several limitations to this study. Firstly, this pilot study involved few number of subjects. However, deviations in serum calcium levels are very less and the low number of subjects in the pilot study is justified. Secondly, we conducted a single-arm study and a comparison was not made with any other commercial calcium carbonate supplement. The absence of a comparator group might not have affected the absorption or efficacy as determined in this study. A third limitation is that the pharmacokinetic study duration was limited to six hours, and therefore complete ΔAUC of serum calcium could not be calculated. However, a similar approach was used previously [27,28]. Our fourth limitation was that we were not able to measure calcium absorption isotopically because commercial formulation cannot be labeled with calcium isotopes.

## Conclusions

The results of this study highlighted that short-term calcium carbonate supplementation is beneficial as it strengthens bone formation and bone density. However, it is imperative to select a product with adequate disintegration, dissolution, and quality. Our results demonstrated that Gemcal DS, one of the popular calcium products, is effective and well-tolerated for short-term use by the healthy population. It produced an appropriate pharmacodynamic response, with a greater suppression of parathyroid function. Nevertheless, long-term studies on large populations are recommended to validate the results.
